# Do We Need New Electrocardiographic Criteria for Left Ventricular Hypertrophy? The Case of the Peguero–Lo Presti Criterion. A Narrative Review

**DOI:** 10.1093/ajh/hpad117

**Published:** 2023-12-19

**Authors:** Andrea Faggiano, Elisa Gherbesi, Marijana Tadic, Stefano Carugo, Guido Grassi, Cesare Cuspidi

**Affiliations:** Foundation IRCCS Ca’ Granda Ospedale Maggiore Policlinico, Milano, Italy; Department of Clinical Sciences and Community Health, University of Milano, Milano, Italy; Department of Clinical Sciences and Community Health, University of Milano, Milano, Italy; Department of Cardio-Thoracic-Vascular Diseases, University Heart Center Ulm, University Ulm, Ulm, Germany; Foundation IRCCS Ca’ Granda Ospedale Maggiore Policlinico, Milano, Italy; Department of Clinical Sciences and Community Health, University of Milano, Milano, Italy; Department of Medicine and Surgery, University of Milano-Bicocca, Milano, Italy; Department of Medicine and Surgery, University of Milano-Bicocca, Milano, Italy

**Keywords:** blood pressure, echocardiography, electrocardiography, hypertension, left ventricular hypertrophy, Peguero–Lo Presti

## Abstract

The cardiovascular risk associated with left ventricular hypertrophy (LVH) in the community and, particularly, in the hypertensive fraction of the general population, represents the rationale for its timely and accurate identification in order to implement adequate preventive strategies. Although electrocardiography (ECG) is the first-line and most economical method of diagnosing LVH its accuracy is largely suboptimal. Over the last 70 years, dozens of different ECG criteria, mostly based on measurements of QRS voltages, have been proposed. In this long journey, a few years ago Peguero *et al.* developed a novel ECG voltage criterion, currently recognized as Peguero–Lo Presti (PLP) suggesting that it has greater sensitivity than traditional ECG-LVH criteria. Considering that in the last 5 years numerous studies have investigated the diagnostic value of this new index, this review aimed to summarize the data published so far on this topic focusing both on the accuracy in identifying the presence of LVH compared with imaging techniques such as echocardiography (ECHO) and magnetic resonance imaging (MRI) and the value in predicting hard outcomes. The evidence in favor of the greater diagnostic accuracy of the PLP criterion in detecting LVH, phenotyped by ECHO or MRI, and in the stratification of hard outcomes compared with traditional ECG criteria does not appear to be sufficiently proven. Given that the diagnosis of LVH by all ECG criteria (including the PLP) exclusively based on the QRS amplitude is largely imprecise, the development of new multiparametric ECG criteria based on artificial intelligence could represent a real improvement in the diagnostic capacity of the ECG.

## Graphical Abstract

**Graphical Abstract:**
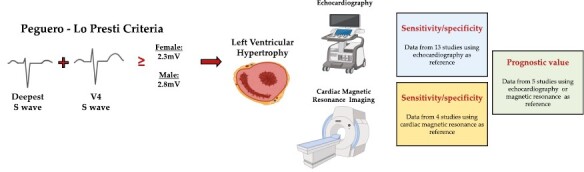

Left ventricular hypertrophy (LVH) represents an independent predictor of increased risk of cardiovascular events, cardiovascular mortality, and all-cause mortality in the general population and a variety of clinical settings.^1–3^ Systemic hypertension is the most important risk factor involved in the transition process from normal structure/geometry to LV remodeling up to LVH by inducing myocyte hypertrophy and interstitial fibrosis resulting in alterations of both LV contractility and relaxation.^4^ LVH reflects an intermediate step in the disease continuum linking established risk factors such as hypertension, dyslipidemia, obesity, and diabetes mellitus, to nonfatal and fatal cardiovascular events.^5,6^ The detection of LVH has long been considered an important diagnostic objective for identifying high-risk patients deserving of targeted therapeutic interventions.^7^ Indeed, the identification of hypertensive-mediated cardiac organ damage is crucial for the proper stratification of the patient’s overall cardiovascular risk. The importance of LVH detection has also increased with the recognition that LVH can regress as a result of therapy and that this can delay or prevent adverse cardiovascular outcomes. In particular, different antihypertensive agents have proven effective in regressing LVH.^8,9^ The diagnostic accuracy in identifying LVH varies in relation to the technique used, being clearly lower with the electrocardiogram (ECG) than with imaging techniques such as 2- or 3-dimensional echocardiogram (ECHO), computerized tomography, and even more so with magnetic resonance imaging (MRI).^4,10^ Although these imaging techniques provide a more accurate assessment of LV mass (LVM) than the ECG, they cannot systematically replace the ECG. Wide availability and low cost are the key factors that make the ECG still not replaceable as the first step in detecting LVH for clinical, epidemiological, and research purposes. Although the ECG is a routinely used screening tool its accuracy in predicting the presence of anatomical LVH, however, is largely suboptimal.^11^ Since the late 1940s, dozens of different ECG criteria mostly based on measurements of QRS voltages have been proposed in order to improve sensitivity and specificity.^12^

In this long journey of research aimed at identifying better performing ECG criteria to diagnose LVH, few years ago Peguero *et al.*^13^ proposed a novel ECG voltage criterion, now currently defined as Peguero–Lo Presti (PLP), based on the sum of the maximum S wave in any lead and the S wave in lead V4. This new criterion tested in that pioneering study in a small sample of patients demonstrated greater diagnostic sensitivity in comparison with Sokolow–Lyon (SL) and Cornell voltage (CV) indices. Since then numerous studies on the topic and a couple of meta-analyses have been published suggesting a greater sensitivity of the PLP compared with SL and CV criteria with, however, a lower diagnostic specificity, leaving open the question of its greater accuracy in detecting LVH compared with traditional criteria.^14,15^ It is worth noting that the confirmation of traditional ECG criteria in the assessment of subclinical cardiac organ damage by the 2023 European Society Hypertension (ESH) guidelines would appear to reflect the lack of definitive evidence in favor of the diagnostic value of PLP.^16^ Starting from these premises we analyzed the results of the studies that compared: (i) sensitivity, specificity, and diagnostic accuracy of the LPL criterion compared with the SL and CV criteria in identifying LVH defined by ECHO or MRI and (ii) its prognostic predictive value for cardiovascular events and/or mortality.

## METHODS

The present article was prepared in accordance with the Narrative Review Checklist (available at http://dx.doi.org/10.21037/jtd-20-2728). The medical literature was reviewed in order to identify all articles comparing the performance of the PLP criterion in identifying LVH to traditional ones using ECHO and MRI as reference standard. A computerized search was performed using Pub-Med, OVID, EMBASE, and Cochrane library databases from inception up to 31 August 2023. Studies were identified by using the following search terms: “Peguero-Lo Presti,” “left ventricular hypertrophy,” “electrocardiography,” “echocardiography,” and “cardiac magnetic resonance imaging.” Checks of the reference lists of selected papers and pertinent reviews complemented the electronic search. Data were examined and extracted by 3 independent investigators (E.G., C.C., and M.T.).

## RESULTS

**Figure 1.**
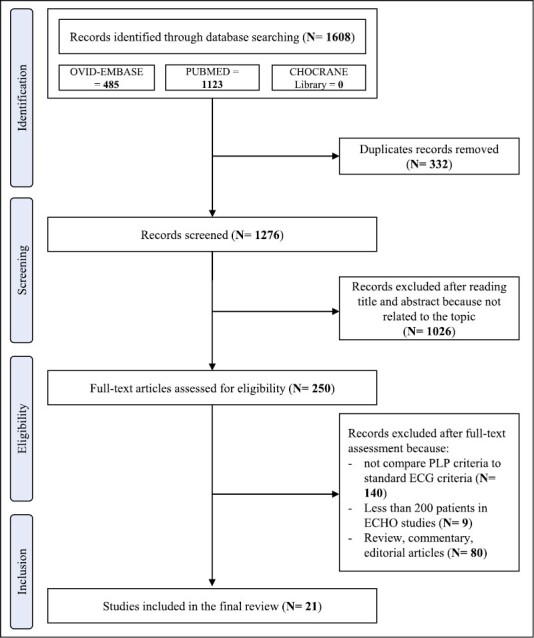


The first literature search identified 1,276 papers. After the initial screening of titles and abstracts, 1,026 studies were excluded as they were not related to the topic. Therefore, 250 studies were reviewed; of these, 140 did not compare the performance of the PLP criterion to standard ECG criteria (i.e., SL or CV) in diagnosing LVH or stratifying cardiovascular prognosis or mortality. Reviews, commentaries, and letters were excluded. As for the studies that tested the diagnostic sensitivity of the various ECG indices of LVH having as reference LVM mass determined by ECHO, we excluded those with fewer than 200 patients. This exclusion criterion was not used for studies in which LVM was determined with MRI due to the paucity of publications on this issue. A total of 21 studies containing sufficient clinical and cardiac imaging data were included in the final review (Figure 1).^13,17–36^ The Newcastle-Ottawa Score, used for assessing the quality of the studies, ranged from 7 to 9, the mean score being 7.7. Therefore, no study was excluded based on its limited quality.

On the whole, 41,030 individuals were included in 21 studies (sample size ranging from 200 to 10,614 participants), performed in 5 continental areas (Asia = 9, Europe = 6, North America = 3, Africa = 2, South America = 1).

### Diagnostic value of PLP in detecting LVH defined by ECHO

Table 1 shows demographic and clinical characteristics of 14 comparative studies targeting the value of PLP criterion vs. established ECG criteria (i.e., SL and CV) in detecting LVH by ECHO. The mean age range was 48–77 years; 46.9% of participants were men. Mean body mass index varied from 22.8 ± 3.0 to 31.1 ± 3.9 kg/m^2^ (data provided by 9 studies). Mean systolic blood pressure (BP) ranged from 126 ± 19 to 154 ± 30 mm Hg, and diastolic BP from 76 ± 11 to 95 ± 16 mm Hg. The majority of studies included patients referred to ECHO labs for suspected heart disease and free-living members of the general population; 3 studies were carried out in hospitalized patients, in elderly and in patients with aortic stenosis, respectively, and 2 studies in hypertensive cohorts.

**Table 1.** Demographic and clinical characteristics of 14 comparative studies targeting the value of Peguero–Lo Presti (PLP) criterion vs. established diagnostic electrocardiographic criteria (SL, Sokolow–Lyon voltage; CV, Cornell voltage) in detecting left ventricular hypertrophy (LVH) having echocardiography as reference

Author (reference)	Sample size (*n*)	Age (y)	Sex (% male)	BMI (kg/m^2^)	Blood pressure (mm Hg)	Setting
Peguero^13^	216	61 ± 16	49	n.a.	145 ± 33/82 ± 17	Hospitalized ± patients referred for echocardiography
Patted^17^	400	64 ± 10	73.5	n.a.	144 ± 17/86 ± 8	Hypertension
Sun^18^	10,614	54 ± 10	47.4%	24.8 ± 3.6	141 ± 15/82 ± 9	General Population
Moustafa^19^	200	60 ± 9	79.5	31.1 ± 3.9	130 ± 29/82 ± 11	Patients referred for Echocardiography
Narita^20^	866	55 ± 15	37.0	22.8 ± 3.0	130 ± 19/78 ± 11	General Population
Shao^21^	235	65 ± 10	49.5	n.a.	n.a.	Hypertension
Keskin^22^	767	51 ± 16	47.2	27.0 ± 5.1	n.a.	Patients referred for Echocardiography
Ricciardi^23^	2,134	69 ± 13	48.0	25.9 ± 4.0	n.a.	Hospitalized patients
Chen^24^	10,360	54 ± 11	44.7	24.8 ± 3.6	141 ± 23/82 ± 12	General Population
Luangphiphat^25^	317	57 ± 16	42.3	n.a.	n.a.	Patients referred for Echocardiography
Lv^26^	7,415	48 ± 15	47.1	24.2 ± 3.6	126 ± 19/82 ± 11	General Population
Nyaga^27^	238	58 ± 13	45.8	28.6 ± 5.6	154 ± 30/95 ± 16	Patients referred for Echocardiography
Tavares^28^	592	77 ± 6	49.2	26.3 ± 4.0	133 ± 22/76 ± 11	Elderly
Sager^36^	279	73 ± 10	58	n.a.	n.a.	Aortic stenosis

Data are presented as absolute numbers, percentage, mean ± SD. Abbreviation: BMI, body mass index.

As reported in Table 2, the prevalence of ECHO LVH varied markedly between studies (i.e., 11.6%–58.0%), although it should be emphasized that most of them included samples with a high prevalence of this adverse cardiac phenotype (over 40% in 7 of the 13 studies providing provided this information). The diagnostic sensitivity of the PLP criterion compared with SL and/or CV criteria was greater in 11 out of the 13 studies. A gender-based analysis performed by 2 studies suggested greater PLP sensitivity in both sexes. On the contrary, the diagnostic specificity of the PLP criterion was lower than the traditional ones in almost all the studies considered. Table 3 summarizes the overall diagnostic performance of the 3 ECG criteria assessed by receiver-operating characteristic curves and expressed as area under the curve (AUC). All but one of the 10 studies providing this metric reported that the AUC associated with the PLP was lower than with the CV criterion. This was not the case for the SL criterion whose AUC was lower than that of PLP in the majority of studies.

**Table 2.** Sensitivity and specificity data provided by 13 comparative studies that addressed the value of Peguero–Lo Presti (PLP) criterion vs. established diagnostic electrocardiographic criteria (SL, Sokolow–Lyon voltage; CV, Cornell voltage) in detecting left ventricular hypertrophy (LVH) having echocardiography as reference

Author (reference)	LVH prevalence by ECHO (%)	Sensitivity (%)			Specificity (%)		
		SL	CV	PLP	SL	CV	PLP
Peguero^13^	30.0	17.0	35.0	62.0	98.0	92	90.0
Patted^17^	48.0	29.1	39.6	54.1	86.5	89.4	91.3
Shao^21^	49.3	64 M, 51 F	55 M, 57 F	65 M, 81 F	57 M, 59 F	76 M, 94 F	74 M, 77 F
Sun^18^	–	45 M, 12 F	21 M, 19 F	57 M, 42 F	78 M, 97 F	96 M, 96 F	67 M, 83 F
Moustafa^19^	41.5	26.5	32.5	55.4	92.3	97.4	83.0
Narita^20^	18.0	24.0	42.0	21.0	89.0	89.9	94.0
Ricciardi^23^	58.0	24.8	31.1	42.3	91.6	88.8	75.8
Keskin^22^	20.1	3.9	9.7	17.5	97.6	98.2	94.5
Luangphiphat^25^	51.7	18.3	14	29.9	88.9	98.0	90.6
Lv^26^	11.6	12.0	12	29.0	89.0	95.0	73
Nyaga^27^	45.3	48.1	63.9	63.9	83.3	79.2	73.8
Tavares^28^	40.7	28.2	35.3	51.9	92.6	98.7	82.1
Sager^36^	47.0	40.0	47.0	50.0	72.0	85.0	72.0

Data are presented as percentage. Abbreviations: F, female; M, male.

**Table 3.** Summary of 10 comparative studies that reported data on the area under the ROC curves for Peguero–Lo Presti criterion vs. established diagnostic electrocardiographic criteria in detecting left ventricular hypertrophy (LVH) having echocardiography as reference

Author (reference)	Sokolow–Lyon AUC	Cornell voltage AUC	Peguero–Lo Presti AUC
Sun^18^	0.665 M, 0.648 F	0.699 M, 0.721 F	0.665 M, 0.689 F
Narita^20^	0.610	0.740	0.630
Ricciardi^23^	0.614	0.678	0.641
Keskin^22^	0.520	0.670	0.640
Chen^24^	0.670 M, 0.640 F	0.690 M, 0.700 F	0.650 M, 0.660 F
Luangphiphat^25^	0.590	0.710	0.670
Lv^26^	0.510	0.550	0.510
Nyaga^27^	0.652	0.716	0.689
Tavares^28^	0.670	0.660	0.700
Sager^36^	0.590	0.700	0.650

Abbreviations: AUC, area under the ROC curves; F, female; M, male; ROC, receiver-operating characteristic.

### Diagnostic value of PLP in detecting LVH defined by MRI

The prevalence of LVH determined by MRI varied markedly across the 4 studies that compared the value of the PLP criterion in detecting increased LV mass (i.e., 7.2%–77.2%). As reported in Table 4, the sensitivity of the PLP criterion was higher than the CV in all studies, and, in 3 out of 4 compared with the SL criterion. Of note, SL emerged as the most sensitive criterion in the largest study published to date. The specificity of the PLP was lower than that expressed by both CV and SL criteria in 2 studies, and substantially comparable in the other 2 studies.

**Table 4.** Summary of 4 comparative studies that addressed the value of Peguero–Lo Presti criterion vs. established diagnostic electrocardiographic criteria in detecting left ventricular hypertrophy (LVH) having resonance magnetic imaging (RMI) as reference

Author (reference)	Sample (*n*)	LVH prevalence (%) and MRI criteria		Sensitivity (%)			Specificity (%)		
				SL	CV	PLP	SL	CV	PLP
Sparapani^29^	4,714	7.2	LVM/LVM predicted >1.31	21.7	5.8	14.5	94.1	97.2	93.8
Guerreiro^30^	240	62.1	>90 g/m^2^ in men >78 g/m^2^ in women	25.0	29.0	47.0	93.2	94.0	93.5
Liu^31^	138	21.0	83 g/m^2^ in men 67 g/m^2^ in women	59.6	50.5	82.2	82.6	88.0	65.8
Matusik^32^	38	72.2	72 g/m^2^ in men 55 g/m^2^ in women	12.0	4.0	56.0	92.3	100	84.6

Data are presented as absolute numbers and percentages. Abbreviations: CV, Cornell voltage; LVM, left ventricular mass; PLP, Peguero–Lo Presti; SL, Sokolow–Lyon voltage.

### Prognostic value of PLP

As shown in Table 5, 3 large retrospective population-based studies and 2 small cohort studies (carried out in hemodialysis and aortic stenosis settings) investigated the value of the PLP criterion in predicting mortality (i.e., all-cause, cardiovascular mortality, and sudden cardiac death). As for the general population, the first study published on the topic showed no differences in the prognostic value between PLP, SL, and CV. Subsequently, 2 studies reported that CV performed better than PLP and SL when the ECG criteria were treated as dichotomous variables. Among hemodialysis patients the PLP and CV, but not the SL criterion, were independently associated with cardiovascular mortality. Finally, the study conducted in patients with aortic stenosis revealed that PLP, contrary to SL and CV, was independently associated with all-cause mortality.

**Table 5.** Summary of comparative 5 studies that addressed the value of Peguero–Lo Presti (PLP) criterion vs. established electrocardiographic criteria (SL, Sokolow–Lyon voltage; CV, Cornell voltage) in predicting all-cause mortality or cardiovascular events

Author (reference)	Design	Setting	Sample (*n*)	Age (y)	Male (%)	Outcome	Main findings
Afify^33^	Retrospective	PBS	7,825	59.8 ± 13.4	47.3	All-cause and CV mortality	PLP criterion was predictive of increased risk of death similar to the SL and CV criteria.
Porthan^34^	Retrospective	PBS	5,730	52.1 ± 13.1	46.3	SCD	PLP, SL, and CV criteria were associated with SCD risk as continuous variables. As for dichotomous variables only CV was an independent predictor of SCD.
Sparapani^29^	Retrospective	PBS	4,714	61.3 ± 10.1	46.4	All-cause mortality and CV events	HR of all-cause mortality entailed by LVH defined by CV was greater than SL and PLP criteria; while that of cardiovascular and coronary heart disease was slightly higher for PLP than for the other 2 criteria.
Braunisch^35^	Retrospective	HD	308	66.5 (53.2–75.5)	65.6	CV mortality	PLP and CV were independently associated with cardiovascular mortality, this was not the case for SL.
Sager^36^	Retrospective	AS	279	73 ± 10	58	All-cause mortality	PLP criterion was independently associated with all-cause mortality, this was not the case for SL and CV.

Abbreviations: AS, aortic stenosis; BART, Bayesian Additive Regression Trees; CV, cardiovascular; ECG, electrocardiogram; HD, hemodialysis; HR, hazard ratio; LVH, left ventricular hypertrophy; PBS, population-based study; SCD, sudden cardiac death. Data are presented as absolute numbers, percentage, mean ± SD, and interquartile range.

## DISCUSSION

Since 2017 a growing amount of information has accumulated on the value of the PLP criterion for detecting LVH leading to the publication of 2 meta-analyses and the choice of this criterion by a large trial that addressed the effects of intensive BP control in hypertensive elderly both on regression and new onset LVH.^14,15,37^

The first of these meta-analyses based on 10 studies conducted in different clinical settings with a total of 5,984 individuals suggested that the PLP had better sensitivity (43%) compared with CV (26%) and SL (22%) but less specificity with an overall diagnostic accuracy superior to the other 2 criteria (i.e., AUC = 0.83 compared with 0.72 and 0.62 for CV and SL, respectively).^14^ A subsequent meta-analysis, comprising 6 trials and 13,564 patients, confirmed the lower specificity of PLP compared with the traditional criteria and revealed that its diagnostic accuracy was significantly higher (AUC = 0.69) than that of the SL (AUC = 0.28) but only slightly higher than the CV (AUC = 0.67).^15^

The Strategy of Blood Pressure Intervention in the Elderly Hypertensive Patients (STEP) trial compared the effects of intensive antihypertensive treatment (SBP target, 110 to <130 mm Hg) vs. those of standard treatment (130 to <150 mm Hg) on the dynamic changes in LVH, defined by the PLP criterion (sum of the amplitude of the deepest S wave and the S wave in the V4 lead ≥2,300 µV for women and ≥2,800 µV for men) in 7,141 older patients with hypertension.^37^ Intensive SBP lowering was associated with a slower progression of the mean PLP value resulting in reduced risk of new onset LVH (HR = 0.76, CI: 0.66–0.89, *P* < 0.001). The choice of the STEP trial investigators was motivated by the fact that the ECG criteria for diagnosis of LVH, based on measurement of R wave amplitude, are low in sensitivity and that the changes in QRS voltage in patients with mild to moderate LVH are better represented by the S wave.

However, despite emerging evidence in favor of this new criterion, the 2023 European Society Hypertension (ESH) guidelines recommended 4 different traditional criteria, exclusively (i.e., SL and R wave in AVL) or partially based on R wave amplitude (i.e., CV and CV × QRS duration).^16^

Thus, in an attempt to offer an updated contribution to knowledge on the value of the PLP in the diagnosis of LVH compared with traditional ECG criteria, we examined this interesting issue targeting: (i) sensitivity/specificity/diagnostic accuracy of this criterion in studies having as reference LVM assessed by ECHO and MRI; (ii) prognostic capacity in predicting cardiovascular events and mortality.

As regards ECHO studies, in this review we have considered only those with populations of at least 200 cases with the aim of focusing on studies based on sufficiently representative samples and, potentially with a reliable power of discrimination of the performance of the various diagnostic criteria. On the contrary, we did not use the sample size filter for studies that had MRI as the gold standard for LVH, due to the very limited availability of the literature data.

The diagnostic sensitivity of the PLP criterion was higher than the SL in 12 out of 13 studies and in 11 of 13 compared with the CV. On the contrary, its specificity was lower than the SL in 8 out of 13 studies and in 11 out of 13 studies compared with the CV. Regarding diagnostic accuracy, the receiver-operating characteristic curves, of the 3 ECG-LVH scores for the prediction of ECHO LVH, generated by plotting sensitivity over specificity, showed that the CV had the highest AUC in 9 of 10 studies that provided this kind of information. The PLP criterion performed better than the SL in all the studies considered with the exception of that of Chen *et al.*^24^ which highlighted that the SL criterion had a higher AUC in men. The studies included in the present review do not allow to draw indications whether the sensitivity/specificity parameters can be influenced by the prevalence of LVH in a given population, considering that no differences emerged between studies with low (i.e., <20%) compared with those with high LVH prevalence (i.e., >40%).

As for any differences in diagnostic performance in men and women, only a couple of studies have provided a separate analysis by sex,^18,21^ highlighting that the diagnostic accuracy of the PLP criterion was comparable in the 2 sexes. No study analyzed the impact of overweight and obesity on the sensitivity and accuracy of the PLP criterion, and therefore this topic remains undefined so far, although studies conducted in populations with higher body mass index showed findings in line with those obtained in subjects with lower body mass index. The large majority of patients included in the various studies were Asian (>70%) and therefore the results on the diagnostic accuracy of PLP cannot be completely extrapolated to other ethnic groups.

MRI represents the gold standard for validation of other imaging techniques allowing not only an accurate quantification of cardiac structure and function but also tissue characterization.^38^ High costs and limited availability represent the major obstacles to the more widespread use of this method for clinical and research purposes. In fact, MRI, as a reference technique, has been used by very few studies that have investigated the performance of the PLP criterion in detecting LVH. Three small studies involving a total of 416 patients documented a greater diagnostic sensitivity of PLP compared with SL and CV criteria. This finding has not, however, been demonstrated by the largest of the studies published so far that compared the diagnostic performance of a new LVH criterion using a machine-learning technique called Bayesian Additive Regression (Trees BART)-LVH criteria with traditional ECG-LVH criteria, having MRI as the standard, in the participants to Multi-Ethnic Study of Atherosclerosis (MESA).^29^ The BART-LVH criterion (based on combining several ECG and clinical parameters) revealed the highest sensitivity (29.0%), followed by SL (21.7%), PLP (14.5%), and CV (5.8%) without substantial differences in specificity between the 4 criteria.

Data on the prognostic significance of PLP are still limited to a few studies conducted both in general populations and in specific settings such as patients on hemodialysis and aortic stenosis. The risk of all-cause mortality entailed by the PLP criterion was retrospectively investigated by Afify *et al.* 7,825 individuals belonging to the third National Health and Nutrition Examination Survey.^33^ The authors showed that the PLP ECG-LVH was predictive of increased risk of death similar to the traditional ECG-LVH criteria (i.e., SL and CV). In the aforementioned MESA study, the hazard ratio (HR) of all-cause mortality associated with LVH defined by CV was greater than SL and PLP criteria.^29^ In a small study conducted in 279 patients with severe aortic stenosis before aortic valve replacement the PLP score, but not SL and CV, was associated with all-cause mortality.^36^ Data provided by the Health 2000 survey, an epidemiologic study carried out in Finland in 5,730 subjects suggested that PLP, SL, and CV scores were associated with sudden cardiac death when analyzed as continuous variables.^34^ When single LVH criteria were used as dichotomous variables, only CV persisted to be an independent predictor, after adjustments. Among 308 patients on hemodialysis, post-dialysis PLP and CV were independently associated with cardiovascular mortality over a median follow-up of 3 years and this was not the case for SL criterion.^35^ As regards specific outcomes such as the incidence of cardiovascular and coronary disease, the MESA researchers highlighted a slightly greater predictive value of the PLP criterion compared with CV and SL.^29^

In conclusion, the evidence in favor of the greater diagnostic accuracy of the PLP criterion in the identification of subclinical cardiac organ damage such as LVH, phenotyped by ECHO or MRI, and in risk stratification of hard outcomes such as all-cause mortality, fatal and nonfatal cardiovascular events compared with traditional ECG criteria does not appear to be sufficiently proven.

In perspective, further large studies are needed in order to better delineate the diagnostic role of this criterion in detecting the presence of LVH, taking into account important variables that have not yet been well focused on, such as sex, age, ethnicity, body size, and comorbidities. In this regard, however, it is worthy of note that the diagnosis of LVH exclusively based on the amplitude of the QRS voltage represents a largely imprecise pathophysiological approach. It has been shown that, in addition to increases in QRS amplitude, many other ECG changes are associated with LVH including QRS duration, changes in QRS vectors, abnormalities in the ST segment, in T and P waves.^11^ On that physiopathological basis, the advent of high-frequency acquisition of digital ECG signals and processes relying on artificial intelligence for analyzing multiple ECG parameters with the implementation of clinical variables will make possible the development and implementation in clinical practice of new, more accurate ECG-LVH criteria allowing to overcome the limits of traditional diagnostic criteria based on the simple evaluation of the QRS amplitude.^39^

## Data Availability

Non applicable, the paper is a review.
